# An Atypical Presentation, Diagnostic Challenges, and Treatment Response of a Malignant Small Round Cell Tumor in the Lumbo‐Inguinal Region of an Infant

**DOI:** 10.1002/ccr3.72869

**Published:** 2026-06-07

**Authors:** George Evele, Kouya Francine, Ngock George, Richard Bardin

**Affiliations:** ^1^ Paediatric Oncology Unit/Mbingo Baptist Hospital Mbingo Cameroon; ^2^ Paediatric Surgery Unit/Mbingo Baptist Hospital Mbingo Cameroon; ^3^ Pathology Unit/Mbingo Baptist Hospital Mbingo Cameroon

**Keywords:** complete remission, diagnostic uncertainty, extrarenal Wilms tumor, immunohistochemical analysis, infant, malignant small round cell tumors

## Abstract

Malignant small round cell tumors are a heterogeneous group of aggressive neoplasms characterized by small, round cell morphology. The undifferentiated cells in these tumors exhibit a high nuclear‐cytoplasmic ratio, and definitive histologic diagnosis via light microscopy can be extremely challenging. Diagnostic accuracy significantly improves with immunohistochemistry, molecular analyses, and cytogenetic studies, enhancing diagnosis and treatment, and ultimately leading to better survival rates. Unfortunately, these tests are often not readily available in low and middle‐income countries, where childhood cancer rates are highest. We present a case of a male infant who developed a progressive ulcerative mass over a giant congenital melanocytic naevus in the lumbosacral region. Immunohistochemical analysis of the ulcerative mass was positive for Oscar keratin. Despite the high diagnostic uncertainty, the working diagnosis was extra‐renal Wilms' Tumor with loco‐regional metastasis based on the positive Oscar Keratin. Following the SIOP protocol for Wilms' Tumor management, he underwent neoadjuvant chemotherapy, surgery, and adjuvant chemotherapy. He achieved complete remission with multimodal treatment and is alive 2 years after treatment.

## Key Clinical Message

Childhood cutaneous neoplasms may show red flags like fast growth, firmness, ulceration, fixation, and a diameter over 3 cm. Malignant small round cell tumors pose a great diagnostic challenge in the pediatric population. Extra‐renal Wilms' tumor should be considered in children with an inguinal mass and associated red flag signs.

## 1 Introduction

Malignant small round cell tumors (MSRCTs) are a diverse group of aggressive cancers that differ in cell lineage, genetics, and clinical characteristics [1, 2]. They make up the vast majority of childhood cancers [2]. Every year, approximately 400,000 children are diagnosed with cancer globally [3]. However, about 90% of these children live in low and middle‐income countries where the 5‐year net survival is about 10%–60%, unlike their high‐income counterparts of more than 80% [4].

Malignant small round cell tumors comprise morphologically similar undifferentiated cells with a high nuclear‐cytoplasmic ratio, and their hyperchromatic nuclei stain blue on hematoxylin and eosin [2]. Their undifferentiated feature limits the diagnosis of their histologic origin in light microscopy, but improves with immunohistochemistry, molecular and cytogenetic studies [1]. When complemented with immunohistochemical analysis, the diagnostic accuracy of the hematoxylin and eosin stain improves to about 90% [5]. In a small percentage of malignant round‐cell tumors, achieving a definitive diagnosis remains a challenge, affecting treatment and survival rates [1]. Examples of malignant small round cell tumors are nephroblastoma, non‐Hodgkin lymphoma, neuroblastoma, hepatoblastoma, retinoblastoma, rhabdomyosarcoma, and Ewing's sarcoma [1, 2].

We present the case of a malignant small round cell tumor in an infant with an atypical clinical presentation in the lumbo‐inguinal region's subcutaneous tissue, which posed diagnostic and therapeutic challenges. However, it responded to Wilms' tumor treatment protocol of the International Society of Pediatric Oncology (SIOP) adapted for resource‐limited settings [6].

## 2 Case Presentation and Investigations

We present a male infant with a giant hyperpigmented birthmark in the lumbar and gluteal regions. He is the second child, with no family history of cancer, and his mother had a negative HIV serology during the pregnancy.

**FIGURE 1:**
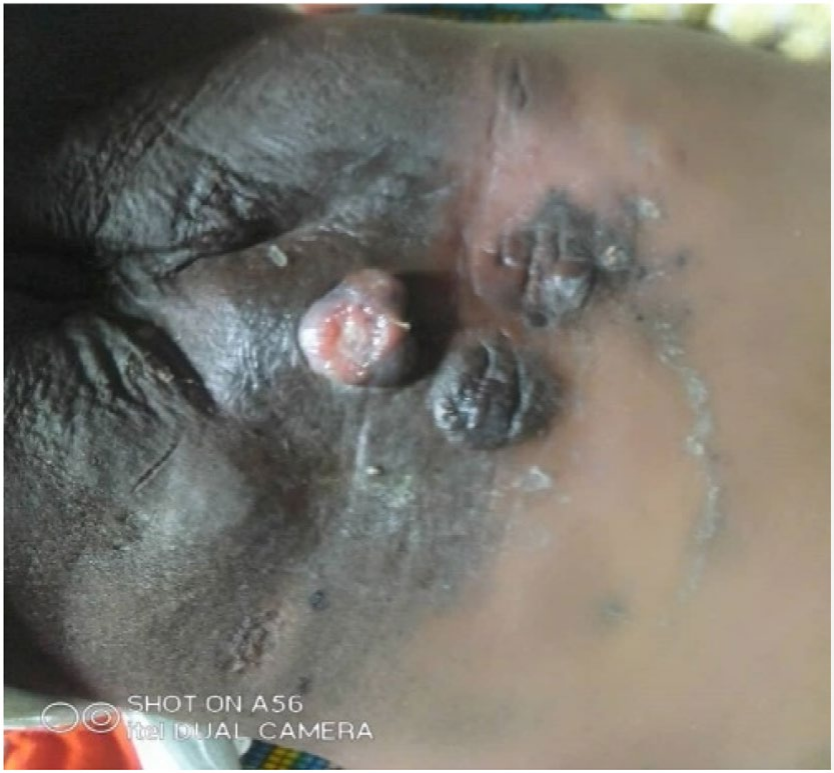
Nodular swellings in the lumbosacral region over a giant birthmark.

At about 1 month of life, the infant developed a gradual onset of swelling over the hyperpigmented birthmark on his lower back, and multiple swellings cropped up by the third month in the same region (Figure 1).

At 5 months old, the mother consulted at a tertiary hospital, where a myelomeningocele was suspected. A cranial magnetic resonance imaging (MRI) was done and showed no abnormalities for his age. An incisional biopsy of the lumbar mass revealed a poorly differentiated small cell tumor of the skin on histopathological analysis. There was no definitive diagnosis, and just supportive care was administered.

**FIGURE 2:**
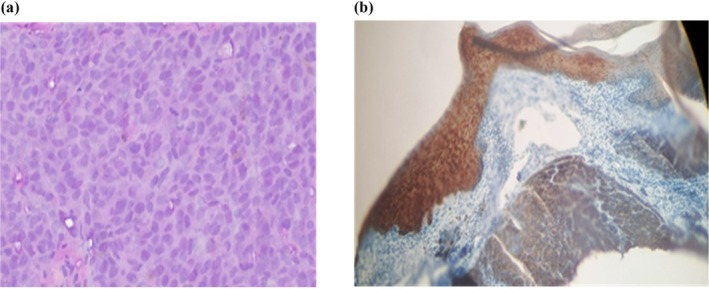
(a) Malignant round blue cell tumor with areas of melanin pigmentation after hematoxylin and eosin stain. (b) A positive Oscar keratin stain is shown by the brown stain in the epidermis and dermis.

At 6 months old, the lower back mass had ulcerated, prompting the mother to self‐refer to our pediatric surgical unit in June 2023 for further evaluation. There, another incisional biopsy of the lumbosacral mass was performed. The histopathology analysis showed a mitotically active tumor. Diffuse proliferation of round cells of medium size with high nuclear‐to‐cytoplasm ratios and a mitotic count of 24/10 high‐power fields (HPFs). The tumor had sheets with thin fibrous septa separating groups of cells. Nuclei were ovoid to round with anisonucleosis. There was a brown pigment present in the epidermis and the tumor. The dermis was filled with a hypercellular small round blue cell tumor with areas of melanin pigmentation (Figure 2a). Since there was no clear histopathological diagnosis, the specimen was sent for immunohistochemical analysis. The tumor was positive for Oscar keratin (Figure 2b) and negative for other stains (Table 1). The changes were in keeping with a malignant round cell neoplasm with epithelial cell differentiation, but no definitive diagnosis.

**TABLE 1:** Results of immunohistochemical analysis of the lumbosacral mass.

Positive stain	Negative stains
Oscar keratin	S‐100
	Desmin
	Leucocyte common antigen
	Chromogranin and Synaptophysin
	Wilms' Tumor Protein 1 marker (WT1)

**FIGURE 3:**
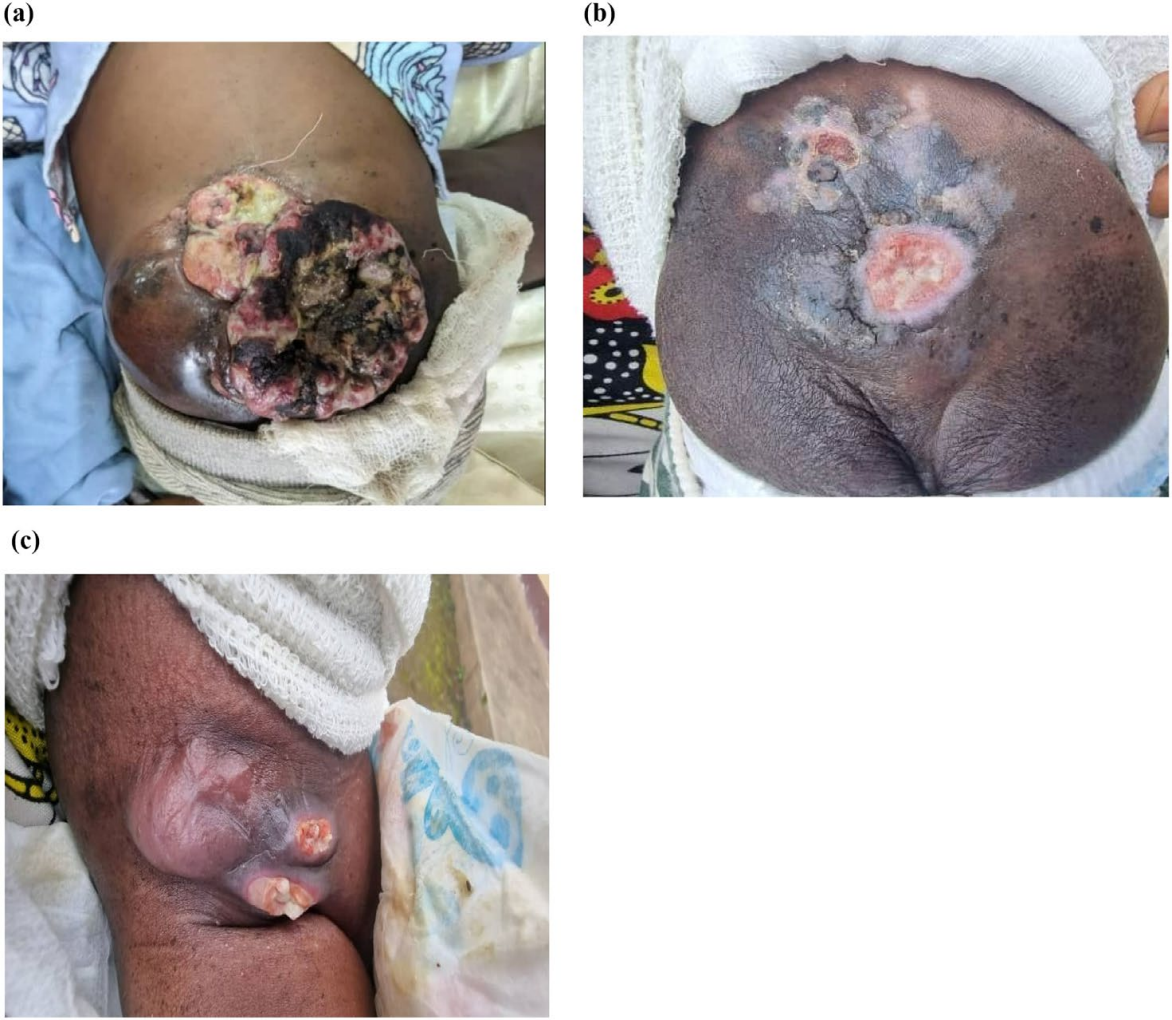
(a) Necrotic ulcerative tumor mass in the lumbosacral region. (b) Residual disease in the lumbosacral region at the end of neoadjuvant chemotherapy. (c) Residual disease in the right inguinal region at the end of neoadjuvant chemotherapy.

About the eight month, the mass was associated with pain and deemed unresectable. The patient was referred to the pediatric oncology unit. He showed signs of clinical anemia and moderate malnutrition. Lung auscultation was clear, and the heart exam revealed regular tachycardia with no added heart sounds. The abdominal exam was benign. His skin was Fitzpatrick phototype VI, with an extensive hyperpigmented patch extending from the lumbar to both gluteal regions. He had multiple necrotic and ulcerative masses with elevated borders in the lumbosacral region (Figure 3a). In addition, he had bilateral inguinal masses from the anterior superior iliac spine to the pubic symphysis, with intact skin and bilateral scrotal swelling displaying positive translucency. The clinical suspicion was advanced soft tissue sarcoma with a differential diagnosis of metastatic congenital melanoma.

Blood tests revealed a white cell count of 41.07 × 10^3^/μL, neutrophilia of 34,436/μL, severe microcytic hypochromic anemia at 6.2 g/dL, and thrombocytosis of 667,000/μL. Electrolytes and renal function were normal, and a malaria smear was negative. Liver function tests indicated elevated alkaline phosphatase (ALP) at 505 U/L, with other results normal. A peripheral blood smear showed neutrophilic leucocytosis, severe microcytic hypochromic anemia, and thrombocytosis, and it was negative for malignant cells. On admission, the abdominal ultrasound and thoracolumbar radiograph showed normal findings.

### 2.1 Management and Response

He was transfused with 140 mL of compatible whole blood, had oral morphine for pain control and wound care. The case was reviewed at the pediatric oncology multidisciplinary team meeting, leading to a diagnosis of probable extra‐renal Wilms' tumor with metastasis to both inguinal regions, supported by a positive Oscar keratin stain seen in nephroblastoma and epithelial tumors. The SIOP Africa Collaborative Wilms Tumor guideline was used, and two‐thirds of the dose was administered, since his weight was < 12 kg. He received alternating combinations of Doxorubicin (30 mg/m^2^), Dactinomycin (45 μg/kg), and Vincristine (1.5 mg/m^2^) weekly, completing 10 cycles as neoadjuvant treatment. After neoadjuvant chemotherapy, the lumbar mass showed an almost complete response (Figure 3b); however, there were residual inguinal ulcerative masses (Figure 3c). A control abdominal ultrasound was done and showed liver, kidneys, spleen, pancreas, and gallbladder were normal. However, the bilateral complex masses in the inguinal regions exhibited an irregular hypoechoic centre and a heterogeneous wall, measured 2.25 × 0.76 cm on the right and 2.5 × 1.1 cm on the left. The inguinal iliac vessels were normal and behind the masses, with no communication noted. A few inguinal lymph nodes were visible in the right inguinal region, the largest measuring 1.2 × 0.5 cm. He had an uneventful bilateral wide local excision of the inguinal masses and lymph nodes under general anesthesia. Histopathological analysis of the left and right groin masses and lymph nodes revealed fibrosis, calcification, necrosis and histiocytes. Reactive lymphoid hyperplasia involving the lymph nodes, and no viable tumor was identified. Finally, he completed 6 cycles of adjuvant chemotherapy comprised of 1 cycle of vincristine 2.0 mg/m^2^ and 5 cycles of doxorubicin 30 mg/m^2^, dactinomycin D 45 μg/kg, and vincristine 2.0 mg/m^2^ combination. Adverse chemotherapy events noted during the course of treatment were Grade 3 neutropenia, Grade 2 alopecia and Grade 1 hyperpigmentation of palms and nails.

### 2.2 Outcome and Follow‐Up

**FIGURE 4:**
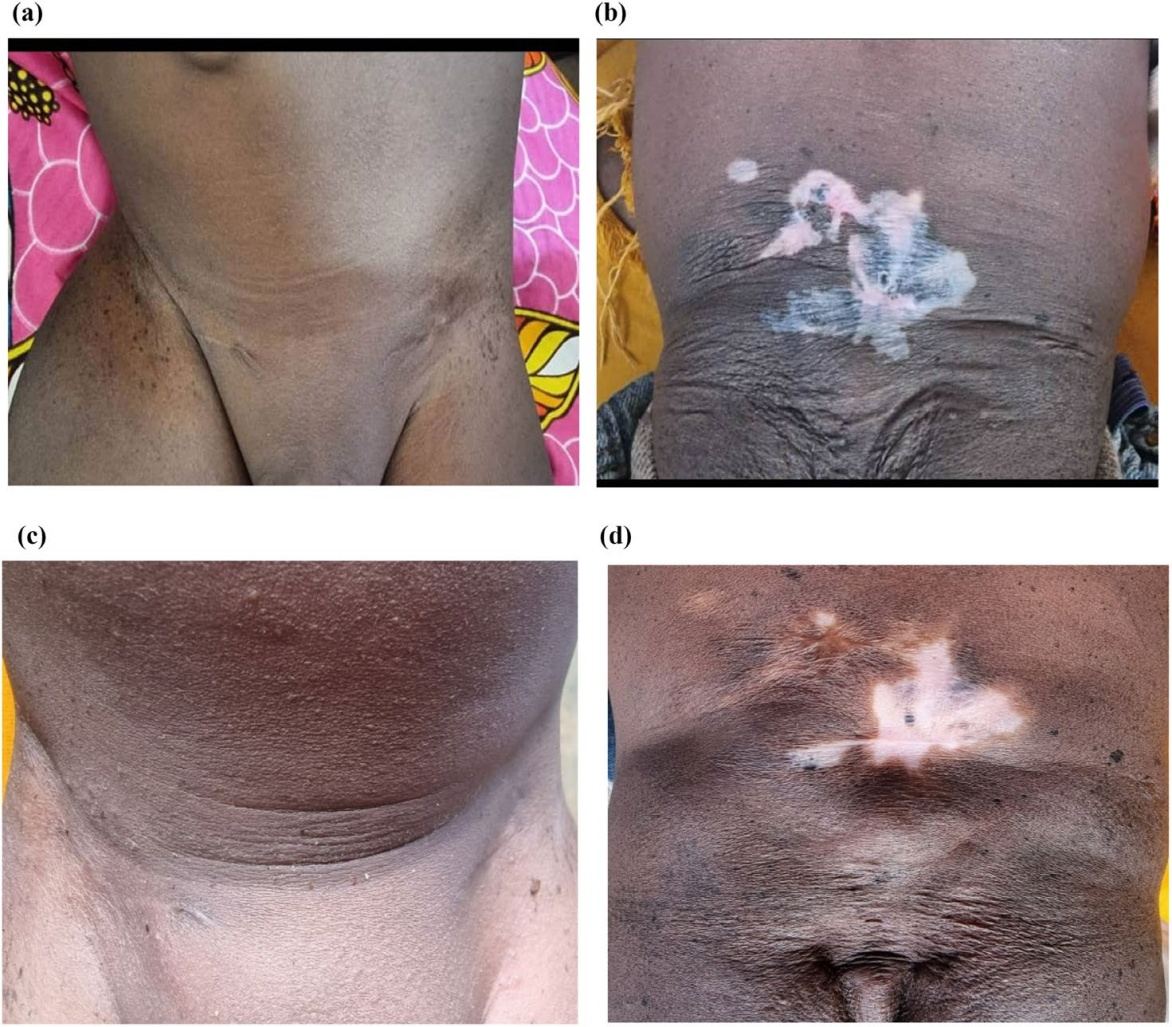
(a) The inguinal regions were clinically free of disease 2 months after completion of treatment. (b) Hyperpigmented and hypopigmented areas in the lumbosacral region at 2 months' follow‐up. (c) Complete clinical response in the inguinal regions at 1‐year follow‐up. (d) Complete clinical response in the lumbosacral region at 1‐year follow‐up.

The patient was reviewed at 2 months after completion of treatment and was doing well. The inguinal regions and lumbosacral region were free of disease (Figure 4a,b).

At 1‐year completion of treatment, there was complete clinical remission at the lumbosacral and both inguinal regions (Figure 4c,d). He is alive in 2026, more than 2 years into complete remission, showing no signs of cardiotoxicity or other chemotherapy‐related adverse events.

## 3 Discussion

The case is an infant with a giant congenital melanocytic naevus in the lumbo‐gluteal region, with rapidly progressive ulcerative swelling in the lumbo‐inguinal region. He was admitted with advanced disease, severe anemia, moderate malnutrition, and elevated alkaline phosphatase. It is worth noting that an MRI of the head, abdominal ultrasound, and radiographs of the chest and thoracolumbar spine were not suggestive of disease involvement. There is a positive correlation between birthmarks and an increased risk of childhood cancer, which we believe is manifested in this patient [7]. In some neoplasms, there is a significant association between high levels of serum alkaline phosphatase and heavy tumor burden, high‐grade tumor, increased risk of metastasis, lymph node invasion, and poor prognosis [8]. This association was strongly expressed in this patient. The high mitotic count of 24/10 per high‐power field was consistent with the tumor's aggressive clinical presentation.

Malignant round cell tumors are a heterogeneous group of malignant neoplasms comprising round cells which are slightly larger or double the size of red blood cells in air‐dried smears or measure less than 10 μm in diameter in alcohol‐fixed smears [2]. Similarly, they have a high nuclear‐to‐cytoplasmic ratio and are relatively undifferentiated, which impairs specific identification based only on morphological features [2]. Some morphological characteristics facilitate the classification of these tumors, namely: based on the round cell pattern, they are grouped into diffuse round cell pattern, septated or lobulated, alveolar or pseudo‐alveolar, round cell pattern with rosettes, haemangiopericytoma‐like vascular pattern, and round cell pattern with other components such as pseudo‐glands and cartilage [9]. Based on the size of the round cells, they are divided basically into small and large round cells. They can be classed based on their cellular origin into neurogenic, mesenchymal, hematolymphoid, and malignant soft tissue tumors of uncertain type [9]. This patient had a malignant small round cell tumor with what seems to be a mixed pattern of diffuse round cell pattern and septated pattern. Although the histologic patterns are nonspecific, they are characteristic of some tumors. For example, the diffuse round cell pattern is seen in lymphoma, malignant melanoma, embryonal rhabdomyosarcoma, and Ewing's sarcoma family of tumors [9]. Also, the septated round cell pattern, which has cells arranged in sheets and separated by fibrous septae, is common in alveolar rhabdomyosarcoma and Ewing's sarcoma family of tumors [9]. Consequently, based on the clinical presentation, a presumptive diagnosis of congenital malignant melanoma or soft tissue sarcoma was proposed. In this context, the atypical clinical presentation and nonspecific histopathologic diagnosis posed a huge challenge to the management of this patient. This supports the fact that the majority of malignant small round blue cell tumors pose significant diagnostic challenges with light microscopy alone. However, further ancillary tests such as immunohistochemistry, molecular analysis, and cytogenetic tests are essential to determine the specific diagnosis and aid with the therapeutic modalities [1].

The Oscar keratin stain produced a positive result for the patient. Oscar keratin is a pan‐cytokeratin antibody that is highly sensitive to tumors of epithelial cell origin [1, 2]. The following stains tested negative for S‐100, Desmin, CD45 (CD: cluster of differentiation) antigen, or leucocyte common antigen (LCA), Chromogranin, Synaptophysin, and WT‐1.

S‐100 is a tumor marker that has more than 95% sensitivity for both primary and metastatic malignant melanoma [1]. A negative result reduces the likelihood of congenital melanoma, even with the presence of a giant melanocytic naevus and melanin pigments. The LCA (CD45) has high sensitivity and specificity for neoplasms of hematolymphoid lineage [1]. A negative stain significantly decreases the likelihood of lymphoma as a diagnosis. Desmin is a marker associated with rhabdomyosarcoma, and its negative result reduces the likelihood of a diagnosis of soft tissue sarcoma. Chromogranin and synaptophysin are nonspecific tumor markers for neuroblastoma; their negativity decreases the likelihood of this diagnosis. Most cases of congenital neuroblastoma occur in the abdominal cavity [1, 9]. A positive pancytokeratin is seen in most carcinomas and Wilms' tumors [1]. Malignant epithelial skin tumors, such as cutaneous squamous cell carcinoma, are extremely rare in childhood [10]. Cutaneous squamous cell carcinoma is not classified as a malignant round cell neoplasm, even though it can test positive for cytokeratins like some MRSTs [9, 11]. The risk factors for cutaneous squamous cell carcinoma include light skin, prolonged exposure to ultraviolet light, and genodermatoses, especially xeroderma pigmentosum [10]. The red flags of cutaneous lesions in childhood include fast growth, firm consistency, spontaneous ulceration, fixed lesions, irregular lesions with rough texture, and a maximum diameter exceeding 3 cm [10]. Our case had all these warning signs suggestive of malignancy.

Our working diagnosis was extrarenal Wilms' tumor with metastasis to both inguinal regions, though the inguinal masses could have been a synchronous primary. Extrarenal Wilms' tumor occurs in about 0.5%–1% of all diagnosed Wilms' tumors, and it affects other sites of the body besides the kidneys [12]. In addition, it is a differential diagnosis to consider in children with inguinal masses with neoplastic features [12]. Our patient had bilateral inguinal masses which were rapidly increasing in size; however, they regressed with the use of cytotoxic agents. Nonetheless, nephroblastoma exhibits a triphasic histologic pattern composed of epithelial, mesenchymal (stromal), and blastemal cells, and the absence of any of these makes the diagnosis of nephroblastoma unlikely in both renal and extrarenal disease [1, 2, 12]. Our case had a positive pancytokeratin, a marker for epithelial differentiation, as is seen in cases of nephroblastoma [1, 9]. However, further molecular analysis will be needed to determine if the epithelial differentiation relates to nephroblastoma. Armanda et al. reported a case of a 1‐month‐old female infant with an extrarenal tumor affecting the subcutaneous tissue of the lumbosacral region [13]. This bears similarities with our case except for the absence of a triphasic pattern and WT‐1 protein nuclear expression. However, the WT‐1 expression is positive in only 25% of extrarenal nephroblastoma, and it is not specific to Wilms' tumor [1, 12]. Furthermore, the management of renal and extrarenal Wilms tumors is the same [13]. Our patient had a complete response after multimodality interventions using the SIOP guidelines for the management of metastatic Wilms' tumor in resource‐limited settings [6]. Anaplasia, a mark of poor prognosis, is uncommon in extrarenal Wilms tumor, resulting in a low disease recurrence rate of about 11% [12]. The 2‐year survival rate for extrarenal Wilms' tumor is approximately 85%, with a mortality rate of about 5% [12].

## 4 Conclusion

Diagnosing certain malignant round cell tumors can be challenging, and their presentation may be atypical. Managing cases without a clear phenotype in developing countries leads to poor outcomes, highlighting the importance of multidisciplinary team discussions to guide management effectively. A patient is never lost; even in suboptimal conditions, there are ways to offer the best palliative care. Precision oncology deserves greater attention to enhance childhood cancer outcomes in developing countries.

### 4.1 Limitations of the Case Report

Genomic sequencing aids in understanding tumor biology and mutations of Wilms' tumors that lack a clear phenotype. This was not performed for our patient as it is unavailable at our institution. However, further ancillary tests are needed for a definitive diagnosis. Although he responded to treatment, the long‐term outcome is not available.

### 4.2 Strengths of the Case Report

Immunohistochemical testing is essential to eliminating the differential diagnosis in malignant round cell tumors that pose a diagnostic challenge. The specimen was reviewed by two pathologists, who confirmed the malignant round cell tumor diagnosis. The case was discussed in a pediatric oncology multidisciplinary team. The SIOP guidelines for Wilms' tumor management in resource‐limited settings effectively improved the patient's outcome.

## Author Contributions

**George Evele:** conceptualization, data curation, investigation, methodology, supervision, validation, visualization, writing – original draft, writing – review and editing. **Kouya Francine:** methodology, writing – review and editing. **Ngock George:** methodology, writing – review and editing. **Richard Bardin:** investigation, supervision, writing – review and editing.

## Funding

The authors have nothing to report.

## Ethics Statement

Ethical approval was obtained from the Cameroon Baptist Convention Health Services Institutional Review Board with assigned study number IRB2025‐82.

## Consent

The authors declare that written informed consent was obtained from the patient's mother for the publication of this manuscript and accompanying images using the consent form provided by the journal.

## Conflicts of Interest

The authors declare no conflicts of interest.

## Data Availability

The data supporting this study's findings are available upon request from the corresponding author. The consent form and ethical approval documents are available upon request from the corresponding author.
